# Deep Eutectic Solvent Based Microwave and Ultrasound Assisted Extraction of Bioactive Compounds From *Vitex agnus‐castus*: Optimization and Antioxidant/Antibacterial Evaluation

**DOI:** 10.1002/cbdv.71751

**Published:** 2026-09-25

**Authors:** Buse Ormancı, Özge Demir, Berrak Dumlupınar, Şah İsmail Kırbaşlar, Aslı Gök

**Affiliations:** ^1^ Department of Chemical Engineering Faculty of Engineering Istanbul University‐Cerrahpaşa Istanbul Türkiye; ^2^ Faculty of Pharmacy Istanbul Okan University Tuzla Istanbul Türkiye

**Keywords:** ABTS, CUPRAC, DPPH, HPLC, optimization, *Vitex agnus‐castus*

## Abstract

This study aimed to optimize microwave‐assisted extraction (MAE) and ultrasound‐assisted extraction (UAE) of bioactive compounds from the leaves of *Vitex agnus‐castus* using deep eutectic solvents (DES). Response surface methodology (RSM) was applied to determine optimal extraction conditions. ChCl:TetEG:H_2_O was selected as the optimal DES for MAE, while ChCl:TriEG:H_2_O was identified as the most suitable solvent for UAE. The physicochemical properties of DES, including FTIR spectra, viscosity, pH, and density, were characterized. Optimal MAE conditions were predicted as a microwave power of 293 W, an extraction time of 119 s, and a liquid‐to‐solid (L/S) ratio of 25.12, whereas optimal UAE conditions were 501.96 W, 23.27 min, and an L/S ratio of 70.08. Antioxidant activities (TAC, DPPH, and ABTS) of DES extracts were compared with those obtained using 70% ethanol under optimal MAE and UAE conditions. Phenolic and flavonoid compounds were quantified by HPLC, including caffeic, syringic, *p*‐coumaric, and ferulic acids, as well as rutin, myricetin, quercetin, and kaempferol. In addition, the antibacterial activities of the extracts were evaluated. The results demonstrate the effectiveness of optimized MAE and UAE combined with DES for the comprehensive recovery of bioactive compounds from *Vitex agnus‐castus*.

## Graphical abstract

Deep eutectic solvent‐based microwave‐ and ultrasound‐assisted extraction strategies are optimized for the recovery of antioxidant compounds from *Vitex agnus‐castus*. The optimized extracts exhibit promising antioxidant and antibacterial activities, while HPLC analysis reveals valuable phenolic compounds. The findings highlight the potential of green extraction approaches for efficient recovery of natural bioactive compounds.

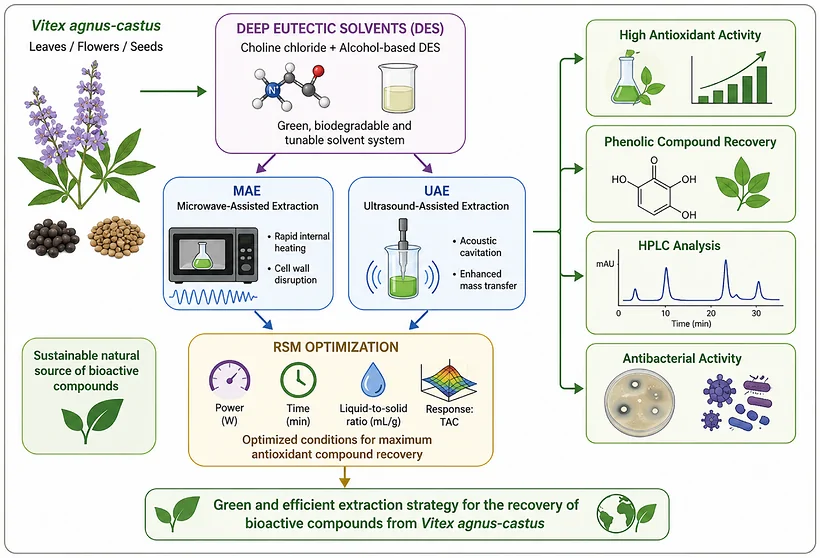

## 1 Introduction

Oxidative stress arises when the balance between oxidant production—such as free radicals and reactive oxygen species—and the body's antioxidant defense systems is disrupted, leading to cellular damage and contributing to the development of various chronic diseases. Antioxidants, including both enzymatic and non‐enzymatic compounds such as vitamins C and E, polyphenols, flavonoids, and carotenoids, play a crucial role in neutralizing these reactive species and protecting biomolecules from oxidative damage. These protective compounds can be synthesized endogenously or obtained through the diet, particularly from fruits and vegetables. Adequate antioxidant intake is therefore essential for cellular protection and for reducing the risk of numerous diseases [1, 2].

*Vitex agnus‐castus*, commonly known as the chaste tree, has long been used in traditional medicine, particularly in the management of women's health conditions. While the fruit has been the primary focus of traditional applications, the leaves have recently attracted increasing scientific interest due to their high concentrations of antioxidants, including flavonoids and phenolic compounds. These bioactive constituents contribute to the neutralization of free radicals and may protect cells against oxidative stress‐induced damage. Studies have demonstrated that the leaves exhibit stronger antioxidant activity than the fruits and contain a diverse range of biologically active compounds. In addition, the leaves possess anti‐inflammatory and hormone‐regulating properties, which support their traditional use in alleviating menstrual disorders. Owing to these beneficial characteristics, *Vitex agnus‐castus* leaves are considered a promising natural source for the development of novel health products and dietary supplements [3, 4, 5, 6, 7].

Modern extraction techniques, such as microwave‐assisted extraction (MAE) and ultrasound‐assisted extraction (UAE), are distinguished by their rapid processing times, high efficiency, and environmentally friendly nature. MAE employs microwave energy to rapidly disrupt plant cell walls, thereby facilitating the release of valuable phytochemicals, whereas UAE utilizes ultrasonic waves to generate cavitation bubbles that enhance the extraction of bioactive compounds [8, 9, 10, 11, 12, 13, 14, 15]. The use of deep eutectic solvents (DES), particularly choline chloride‐based systems, provides additional advantages, as these solvents are cost‐effective, nontoxic, biodegradable, and capable of forming strong hydrogen‐bonding networks that improve extraction efficiency and compound stability [16]. The integration of MAE or UAE with DES has been shown to significantly increase the yield of antioxidants and other bioactive compounds, offering a more sustainable and innovative alternative to conventional extraction methods.

This study evaluated the antioxidant and antimicrobial properties of *Vitex agnus‐castus* leaves collected in Turkey. To the best of our knowledge, this is the first study reporting the use of DES‐based MAE and UAE techniques for the extraction of phenolic compounds from *Vitex agnus‐castus*, whose leaves are considered a promising natural source of bioactive compounds due to their traditional medicinal use and notable antioxidant and antimicrobial potential. Six different DES were prepared using choline chloride in combination with various alcohols, and the most effective solvent was selected based on total antioxidant capacity (TAC) as determined by the CUPRAC assay. Subsequently, the extraction conditions for MAE and UAE were optimized using a Box–Behnken experimental design (BBED). Under the optimized conditions, the resulting extracts were analyzed for TAC, and radical scavenging activity using DPPH and ABTS assays. HPLC was employed to identify and quantify individual phenolic and flavonoid compounds, while antimicrobial activity was evaluated using the broth microdilution method. The results demonstrated that the combination of DES with MAE and UAE provides an efficient and environmentally friendly approach for the recovery of antioxidants from *Vitex agnus‐castus* leaves, with the extracts also exhibiting promising antimicrobial activity. Overall, this green extraction strategy shows considerable potential for the development of novel, natural health products.

## 2 Materials and Methods

### 2.1 Reagents and Chemicals

All chemicals used in this investigation were of analytical grade and were purchased from Sigma–Aldrich (Germany). DES employed in the experimental procedures were prepared by combining choline chloride with various alcohols, including ethylene glycol, diethylene glycol, triethylene glycol, tetraethylene glycol, as well as 1,4‐butanediol, and 1,3‐propanediol.

A range of reagents was used to evaluate antioxidant capacity through different assays. For the CUPRAC assay, copper(II) ions, neocuproine, ammonium acetate, and Trolox were utilized. The DPPH assay employed the DPPH radical, methanol, and Trolox, while the ABTS assay required ABTS salt, potassium persulfate, ethanol, and Trolox.

For chromatographic analysis, standard phenolic and flavonoid compounds—including formic acid, methanol, kaempferol, quercetin, myricetin, ferulic acid, p‐coumaric acid, syringic acid, rutin, and caffeic acid—were used.

### 2.2 DES Preparation Procedure

For the preparation of DES, choline chloride (ChCl) was used as the hydrogen bond acceptor (HBA), while a series of polyols—including ethylene glycol (EG), diethylene glycol (DiEG), triethylene glycol (TriEG), tetraethylene glycol (TetEG), 1,4‐butanediol (1,4‐but), and 1,3‐propanediol (1,3‐pro)—were employed as hydrogen bond donors (HBD). ChCl and each corresponding polyol were accurately weighed (± 0.001 g) using a precision analytical balance, mixed with distilled water at a concentration of 30% (v/v), and subjected to continuous stirring on a magnetic hot plate at 333.15 K until complete dissolution was achieved, resulting in a clear and homogeneous phase.

The controlled addition of water was intended to reduce solution viscosity and thereby enhance extraction efficiency; however, the water content was limited to 30% to avoid detrimental effects on phenolic compound solubility and DES matrix interactions. The specific compositions of the synthesized DES are presented in Table 1.

**TABLE 1:** The specific compositions of the synthesized DES.

DES	HBA	HBD	Molar ratio (HBA:HBD)	Water content (% v/v)
ChCl:EG:H_2_O	ChCl	EG	1:2	30
ChCl:DiEG:H_2_O	ChCl	DiEG	1:2	30
ChCl:TriEG:H_2_O	ChCl	TriEG	1:2	30
ChCl:TetEG:H_2_O	ChCl	TetEG	1:2	30
ChCl:1,4‐but:H_2_O	ChCl	1,4‐but	1:2	30
ChCl:1,3‐pro:H_2_O	ChCl	1,3‐pro	1:2	30

Subsequently, the density of each DES formulation was determined using an Anton Paar density analyzer at 25°C, and pH values were measured with a Mettler Toledo SevenMulti pH meter. Rheological behavior was evaluated by measuring viscosity over a shear rate range of 1–100 s^−1^ using a TA Discovery Hybrid Rheometer (DHR‐1) operated at 25°C. Functional group analysis and structural characterization of the DES samples were carried out by FTIR spectroscopy, with spectra recorded in the range of 4000–400 cm^−1^ using KBr pellets (sample/KBr ratio of 1:200) on a Cary 630 FTIR spectrometer (Agilent, USA).

### 2.3 The Source and Preparation

Dried leaves of *Vitex agnus‐castus* were purchased from a local herbal market in Muğla, Türkiye, in 2024. The plant material was visually inspected, and any foreign materials or deteriorated portions were manually removed prior to further processing. The cleaned samples were ground using a mechanical grinder and subsequently sieved to obtain a uniform particle size of approximately 500 µm. The processed plant material was stored in vacuum‐sealed polyethylene containers to minimize oxidative degradation and moisture uptake during storage.

### 2.4 UAE Protocol

UAE of *Vitex agnus‐castus* leaf samples were performed using a VCX 750 ultrasonic processor (Sonics and Materials Inc., Newtown, USA). The system was equipped with a probe specifically designed to ensure efficient dispersion of ultrasonic energy within the sample matrix.

For each extraction run, 0.5 g of finely ground *Vitex agnus‐castus* leaves was used. Sonication was carried out at 75% amplitude and a constant frequency of 20 kHz. Ultrasonic pulses were applied with an on‐time of 5 s and an off‐time of 2 s, while the extraction temperature was maintained at 50°C throughout the process. Ultrasonic power was varied within the range of 300–700 W.

Extraction times were investigated in the range of 10–30 min, and the liquid‐to‐solid (L/S) ratio (expressed as mL of solvent per g of plant material) was adjusted between 60 and 100 to optimize TAC. Following sonication, the extracts were centrifuged at 5000 rpm for 15 min using a Nüve NF 200 centrifuge. The resulting supernatants were subsequently filtered through 0.45 µm syringe filters and stored at 4°C until further analysis.

### 2.5 MAE Protocol

MAE was performed using a Milestone NEOS‐GR open‐vessel microwave system. For each experimental run, 0.5 g of finely ground *Vitex agnus‐castus* leaf material was accurately weighed into a 50 mL Erlenmeyer flask, followed by the addition of 20 mL of extraction solvent. The standard extraction conditions were set at a microwave power of 300 W for an extraction time of 2 min.

Optimization experiments were conducted by systematically varying microwave power (200–300 W), extraction time (60–120 s), and the liquid‐to‐solid (L/S) ratio (20–60 mL g^−1^). Upon completion of the extraction process, the mixtures were centrifuged at 5000 rpm for 10 min using a Nüve NF 200 centrifuge. The resulting supernatants were filtered through 0.45 µm syringe filters and stored at 4°C prior to further analyses. Absorbance measurements were carried out using a Varian CARY Bio 100 UV–visible spectrophotometer (Agilent, USA).

### 2.6 Measurement of TAC

TAC of the extracts obtained from the plant material was determined using the CUPRAC assay, as previously described [17]. This method is based on the reduction of the chromogenic copper(II)–neocuproine (Cu(II)–Nc) complex to the copper(I)–neocuproine (Cu(I)–Nc) complex by antioxidants present in the sample. The formation of the yellow–orange Cu(I)–Nc complex leads to an increase in absorbance, which is measured spectrophotometrically at 450 nm [18].

For the assay, 1 mL of 10 mM Cu(II) solution, 1 mL of 7.5 mM neocuproine (Nc) solution, and 1 mL of 1 M ammonium acetate (NH_4_Ac) buffer were added to a test tube. An aliquot (x mL) of the sample extract was then introduced, followed by the addition of (1–x) mL of deionized water to obtain a final volume of 4.1 mL. The reaction mixture was incubated for 30 min at room temperature, after which absorbance was recorded at 450 nm against a reagent blank [19].

The TAC values were expressed as milligrams of trolox equivalents per gram of sample (mg TE/g sample) and quantified using a calibration curve constructed with trolox standard solutions [17]. TAC was calculated according to the following equation:

(1) $$\begin{array}{c} \mathrm{TAC}\;\left(\mathrm{mg}\;\mathrm{TE}/g\;\mathrm{sample}\right)\\ =\frac{A_{s}}{\varepsilon_{\;\mathrm{trolox}}}\;\frac{V_{m}}{V_{s}}\mathrm{Df}\;\frac{V_{\varepsilon }}{\;m}\;\mathrm{MA}\;\mathrm{trolox} \end{array}$$

here, *A*_s_ denotes the absorbance of the sample; *ε*_trolox_ (L/mol) represents the molar absorptivity of trolox; *V*_m_ (mL) is the total assay volume (4.1 mL); D_f_ refers to the dilution factor of the sample; *V*_ε_ (mL) denotes the volume of the extract; *V*_s_ (mL) corresponds to the sample aliquot volume; *m* (g) represents the mass of the sample; and *M*_A trolox_ is the molecular weight of trolox [17].

### 2.7 ABTS Assay for Antioxidant Activity

The antioxidant activity of *Vitex agnus‐castus* leaf extracts were evaluated based on their ability to scavenge ABTS radicals using the ABTS assay. This method relies on the reduction of the chromogenic ABTS•^+^ radical cation by antioxidant compounds present in the extracts. To generate the ABTS•^+^ radical solution, 7.0 mM ABTS was dissolved in 50 mL of distilled water, followed by the addition of potassium persulfate (K_2_S_2_O_8_) to achieve a final concentration of 2.45 mM. The reaction mixture was incubated in the dark at room temperature for 12–16 h to allow complete radical formation.

For the assay, an aliquot (x mL) of the *Vitex agnus‐castus* leaf extract was mixed with (4–x) mL of methanol and 1 mL of the preformed ABTS•^+^ solution, which had been diluted tenfold with ethanol. The mixture was allowed to react for 6 min, after which absorbance was measured at 734 nm using ethanol as the reference. The change in absorbance (Δ*_A_*) was used to determine the ABTS radical scavenging activity and was calculated according to the following equation:

(2) $$\Delta_{A}=A_{\mathrm{ABTS}}\text{--}\left(A_{E}-A_{0}\right)$$

where *A*_ABTS_ represents the absorbance of the ABTS•^+^ solution without sample, *A*_E_ is the absorbance of the extract in the presence of ABTS•^+^, and *A*_0_ is the absorbance of the extract without ABTS•^+^.

The ABTS radical scavenging activity of the *Vitex agnus‐castus* leaf extracts was expressed as milligrams of trolox equivalents per gram of sample (mg TE/g sample), based on a calibration curve prepared using trolox standards [17]. The ABTS scavenging capacity was calculated using the following equation:

(3) $$\begin{array}{ccc} & & \text{The}\;\text{ABTS radical scavenging capacity (mg TE/g sample)}\\ & & \;=\frac{\Delta A}{\varepsilon_{\text{trolox}}}\times \frac{V_{m}}{V_{s}}\times D_{f}\times \frac{V_{E}}{m}\times M_{\text{A trolox}} \end{array}$$

where *ε*_trolox_ (L/mol) denotes the molar absorptivity of trolox; *V*_m_ (mL) is the final reaction volume (5 mL); *V*_s_ (mL) is the sample aliquot volume; *D*_f_ represents the dilution factor; *V*_ε_ (mL) is the volume of extract added; *m* (g) is the mass of the sample; and M_A trolox_ refers to the molecular weight of trolox [17].

### 2.8 DPPH Assay for Antioxidant Activity

The DPPH assay was employed to evaluate the free radical scavenging activity of *Vitex agnus‐castus* leaf extracts. This method is based on the ability of antioxidant compounds present in the extract to donate hydrogen atoms to the stable 2,2‐diphenyl‐1‐picrylhydrazyl (DPPH) radical, thereby reducing it to the non‐radical form, diphenylpicrylhydrazine. This reduction is accompanied by a color change of the DPPH solution from deep violet to pale yellow, which can be monitored spectrophotometrically [20].

For the assay, an aliquot (x mL) of the *Vitex agnus‐castus* leaf extract was mixed with (2 − x) mL of methanol and 2 mL of 0.2 mM DPPH solution in a test tube. The corresponding blank contained 2 mL of methanol and 2 mL of 0.2 mM DPPH solution, giving a total volume of 4 mL. Thus, the total reaction volume was maintained at 4 mL for both the blank and sample mixtures. The reaction mixture was allowed to stand in the dark at room temperature for 30 min, after which the absorbance was measured at 517 nm. The decrease in absorbance (Δ_A_), corresponding to the extent of DPPH radical reduction, was used to assess radical scavenging activity [17].

(4) $$\Delta_{A}=A_{\mathrm{DPPH}}\text{--}\left(A_{E}-A_{0}\right)$$

where *A*_DPPH_ represents the absorbance of the DPPH solution in the absence of the sample, *A*_E_ is the absorbance of the extract in the presence of DPPH, and *A*_0_ denotes the absorbance of the extract without DPPH.

The DPPH radical scavenging capacity of the *Vitex agnus‐castus* leaf extracts was expressed as milligrams of trolox equivalents per gram of sample (mg TE/g DS), calculated using a calibration curve prepared with trolox standards. The scavenging capacity was determined according to the following equation:

(5) $$\begin{array}{ccc} & & \text{The}\;\text{DPPH free radical scavenging capacity (mg TE/g sample)}\\ & & \;=\frac{\Delta A}{\varepsilon_{\text{trolox}}}\times \frac{V_{m}}{V_{s}}\times D_{f}\times \frac{V_{E}}{m}\times M_{\text{A trolox}} \end{array}$$

where ε _trolox_ (L/mol) is the molar absorptivity of trolox; *V*_m_ (mL) is the total reaction volume (4 mL); *V*_s_ (mL) is the sample aliquot volume; *D*_f_ denotes the dilution factor; *V*_ε_ (mL) is the volume of extract added; *m* (g) is the mass of the sample; and M_A trolox_ refers to the molecular weight of trolox [17, 19].

### 2.9 HPLC Identification and Quantification

Polyphenol profiling of the analyzed samples was performed using a HPLC system (Agilent Technologies 1260 series) equipped with a reversed‐phase C18 column. Chromatographic separation was achieved using a binary mobile phase system, where solvent A consisted of ultrapure water containing 0.1% formic acid and solvent B consisted of acetonitrile containing 0.1% formic acid. Elution was carried out under a gradient program as follows: 5% B (0–2 min), 15% B (2–27 min), 50% B (27–37 min), 95% B (37–58 min), followed by re‐equilibration to 5% B (58–70 min), at a constant flow rate of 0.2 mL min^−^^1^, as described in [21].

Qualitative and quantitative analyses of individual phenolic acids (*p*‐coumaric, syringic, ferulic, and caffeic acids) and flavonoids (rutin, myricetin, kaempferol, and quercetin) were conducted using the external standard method. Calibration curves for each analyte were constructed over a concentration range of 5–100 ppm. Compound identification and quantification were performed based on their characteristic retention times and detection wavelengths: caffeic acid (310 nm, 21.50 min), syringic acid (280 nm, 22.66 min), p‐coumaric acid (310 nm, 29.21 min), ferulic acid (310 nm, 33.63 min), rutin (254 nm, 35.07 min), myricetin (254 nm, 37.62 min), quercetin (254 nm, 38.94 min), and kaempferol (254 nm, 41.34 min). The linearity of the calibration curves was evaluated using the coefficient of determination (*R* ^2^), with all values exceeding 0.97. The chromatograms are provided in Figure S1. The calibration parameters, retention times, detection wavelengths, calibration ranges, regression equations, and correlation coefficients (*R* ^2^) used for the quantification of phenolic compounds were clearly presented in Table 2.

**TABLE 2:** Calibration parameters and regression equations used for the quantification of phenolic compounds.

Compound name	Detection wavelengths (nm)	Retention time (min)	Calibration ranges (ppm)	Equation	*R* ^2^
Caffeic acid	310	21.5	5–100	*y* = 609.66x+4128.9	*R* ^2^ = 0.98
Syringic acid	280	22.66	5–100	*y* = 179.14x+368.19	*R* ^2^ = 0.99
p‐coumaric acid	310	29.21	5–100	*y* = 579.35x+479.29	*R* ^2^ = 0.99
Ferulic acid	310	33.63	5–100	*y* = 417.64x+567.26	*R* ^2^ = 0.99
Rutin	254	35.07	5–100	*y* = 139.35x+3767.3	*R* ^2^ = 0.97
Myricetin	254	37.62	5–100	*y* = 322.57x+7976.8	*R* ^2^ = 0.99
Quercetin	254	38.94	5–100	*y* = 452.03x+8069.4	*R* ^2^ = 0.99
Kaempferol	254	41.34	5–100	*y* = 366.93x‐234.22	*R* ^2^ = 0.99

### 2.10 Statistical Analysis

The experimental design and regression analyses were conducted using Design‐Expert software (Stat‐Ease Inc., Minneapolis, MN, USA). Differences among experimental groups were evaluated by analysis of variance (ANOVA), with statistical significance defined at *p* < 0.05. Model adequacy and significance were assessed based on the F‐value and *R* ^2^ obtained from ANOVA [16].

For the optimization of MAE, key process variables—including microwave power (200–300 W), extraction time (60–120 s), and the L/S ratio (20–60 mL g^−1^)—were systematically optimized using a BBED within the framework of response surface methodology (RSM). Similarly, UAE parameters, namely ultrasonic power (300–700 W), extraction time (10–30 min), and the L/S ratio (60–100 mL g^−1^), were optimized using the BBED. TAC, expressed as mg trolox equivalents per gram of sample (mg TE/g sample), was selected as the response variable [20].

### 2.11 Antibacterial Activity

#### 2.11.1 Preparation of Extract Dilutions

Extract dilutions were prepared by initially dissolving 320 mg of the dried extract in 1,280 µL of dimethyl sulfoxide (DMSO). From this stock solution, a working solution with a final concentration of 80 mg mL^−1^ was prepared. Serial twofold dilutions were subsequently prepared to obtain extract concentrations ranging from 80 mg mL^−^^1^ to 0.03 mg mL^−1^.

#### 2.11.2 Bacterial Culture

The antibacterial activity of the extracts was evaluated through a series of in vitro assays using standard bacterial strains, including methicillin‐resistant *Staphylococcus aureus* (MRSA, LY 1999 0053), *Klebsiella pneumoniae* ATCC 33495, *Streptococcus pneumoniae* ATCC 49619, and *Pseudomonas aeruginosa* ATCC 27853. All bacterial strains were cultured in accordance with the guidelines of the Clinical and Laboratory Standards Institute (CLSI) to ensure standardized growth conditions [22, 23, 24].

#### 2.11.3 Determination of MIC by Agar Well Diffusion

The minimum inhibitory concentration (MIC) of the extracts was determined by the agar well diffusion method. This approach was selected because the tested extracts exhibited limited aqueous solubility, making the agar well diffusion method more suitable than broth‐based assays for antimicrobial evaluation. Bacterial suspensions were prepared from 24 h agar cultures, washed twice with 0.9% (w/v) saline solution, and adjusted to a final concentration of 1.0 × 10^8^ CFU mL^−1^ using the McFarland standard. An aliquot of 1.66 mL of the standardized bacterial suspension was added to 250 mL of brain–heart agar (BHA), thoroughly mixed, and poured into sterile 9 cm Petri dishes (15 mL per plate). After solidification, wells with a diameter of 6 mm were aseptically punched into the agar and filled with 50 µL of antibiotic solution, extracts, or their combinations. DMSO, Tween 80, and distilled water were used as negative controls. The plates were incubated at 37°C for 24 h, after which the diameters of the inhibition zones were measured in millimeters. The MIC was defined as the lowest extract concentration that produced a visible inhibition zone against the tested microorganism. All experiments were performed in quadruplicate.

## 3 Results and Discussion

### 3.1 DES Selection and Characterization

DES can function as both HBD and HBA, enabling the formation of strong intermolecular interactions with bioactive constituents present in plant matrices. Through these interactions—particularly hydrogen bonding—DES are able to effectively solubilize a wide range of phytochemicals. Previous studies have demonstrated that polyol‐based DES exhibit high extraction efficiencies for phenolic compounds, which can be primarily attributed to the strong affinity between phenolic hydroxyl groups and the functional ─OH and/or Cl^−^ groups within the solvent system [11]. In this work, six alcohol‐based DES systems were prepared using ChCl as the HBA and various polyol‐based HBDs (Table 3). All DES were synthesized at a constant molar ratio of 1:2 (HBA:HBD) and contained 30% (v/v) water to ensure comparable physicochemical conditions during evaluation [25].

**TABLE 3:** Measured viscosity, density, pH, and refractive index of the prepared DES.

Abbreviations	Viscosity (Pa.s)	Density (g/cm^3^)	pH	Refractive index (*n*_D_)
ChCl:EG:H_2_O	0.0202	1.08401	4.422	1.42437
ChCl:DiEG:H_2_O	0.0294	1.09407	4.264	1.4282
ChCl:TriEG:H_2_O	0.0248	1.10093	3.257	1.43282
ChCl:TetEG:H_2_O	0.0261	1.10226	3.419	1.42544
ChCl:1,4‐but:H_2_O	0.0267	1.05201	4.361	1.42996
ChCl:1,3‐pro:H_2_O	0.0334	1.06539	4.487	1.43084

The selection of the most suitable DES was based on its ability to maximize TAC extracted from *Vitex agnus‐castus* leaves. Among the six investigated DES formulations, notable differences in TAC were observed depending on both solvent composition and the extraction technique employed. Under MAE conditions, the ChCl:TetEG:H_2_O system produced the highest TAC values. In contrast, during UAE, the ChCl:TriEG:H_2_O formulation exhibited superior TAC performance.

Based on these results, ChCl:TetEG:H_2_O was selected as the optimal solvent for MAE experiments, while ChCl:TriEG:H_2_O was chosen for UAE studies. Consequently, all subsequent experiments were conducted using these two DES systems, as they provided the most efficient recovery of antioxidant compounds from chaste tree leaves under their respective extraction conditions.

The viscosity, density, pH, and refractive index of the synthesized DES were determined at 25°C to evaluate their suitability for antioxidant extraction. Due to the incorporation of 30% (v/v) water, all DES exhibited viscosity values below 0.1 Pa·s, indicating favorable fluidity and efficient mass transfer during extraction. Slight variations in viscosity were associated with differences in the molecular structure and chain length of the polyol‐based hydrogen bond donors [26]. The density values of all DES were close to that of water and showed only minor differences, reflecting the similar hydroxyl functionalities and identical molar ratios used in their preparation. Refractive index values were found within a narrow range, suggesting comparable polarity and hydrogen‐bonding characteristics among the alcohol‐based DES systems [27]. All synthesized DES exhibited near‐neutral pH values, providing a mild extraction environment that minimizes the risk of degradation of antioxidant compounds. Overall, the physicochemical properties of the selected DES confirm their suitability for MAE and UAE of antioxidants from chaste tree leaves.

To verify the successful formation of DES, comprehensive spectroscopic characterization was performed on both the individual components and the resulting DES systems. This approach enabled the identification of spectral changes associated with variations in intermolecular interactions. Following DES synthesis, noticeable shifts in the characteristic absorption bands of the pure constituents were observed, indicating alterations in the molecular environment and bonding arrangements within the eutectic mixtures [28].

Infrared spectral analysis revealed well‐defined absorption bands corresponding to various functional groups in both the individual components and the synthesized DES, as shown in Figure 1. Broad absorption bands observed in the range of 3200–3550 cm^−1^ were attributed to O─H stretching vibrations, confirming the presence of hydroxyl functional groups and extensive hydrogen bonding. Bands associated with N–H stretching vibrations appeared between 3015 and 3026 cm^−1^, while C─H stretching vibrations of aliphatic and aromatic moieties were detected in the 2800–3000 cm^−1^ region.

FIGURE 1FTIR spectra of DES and their individual components.

**Figure:**
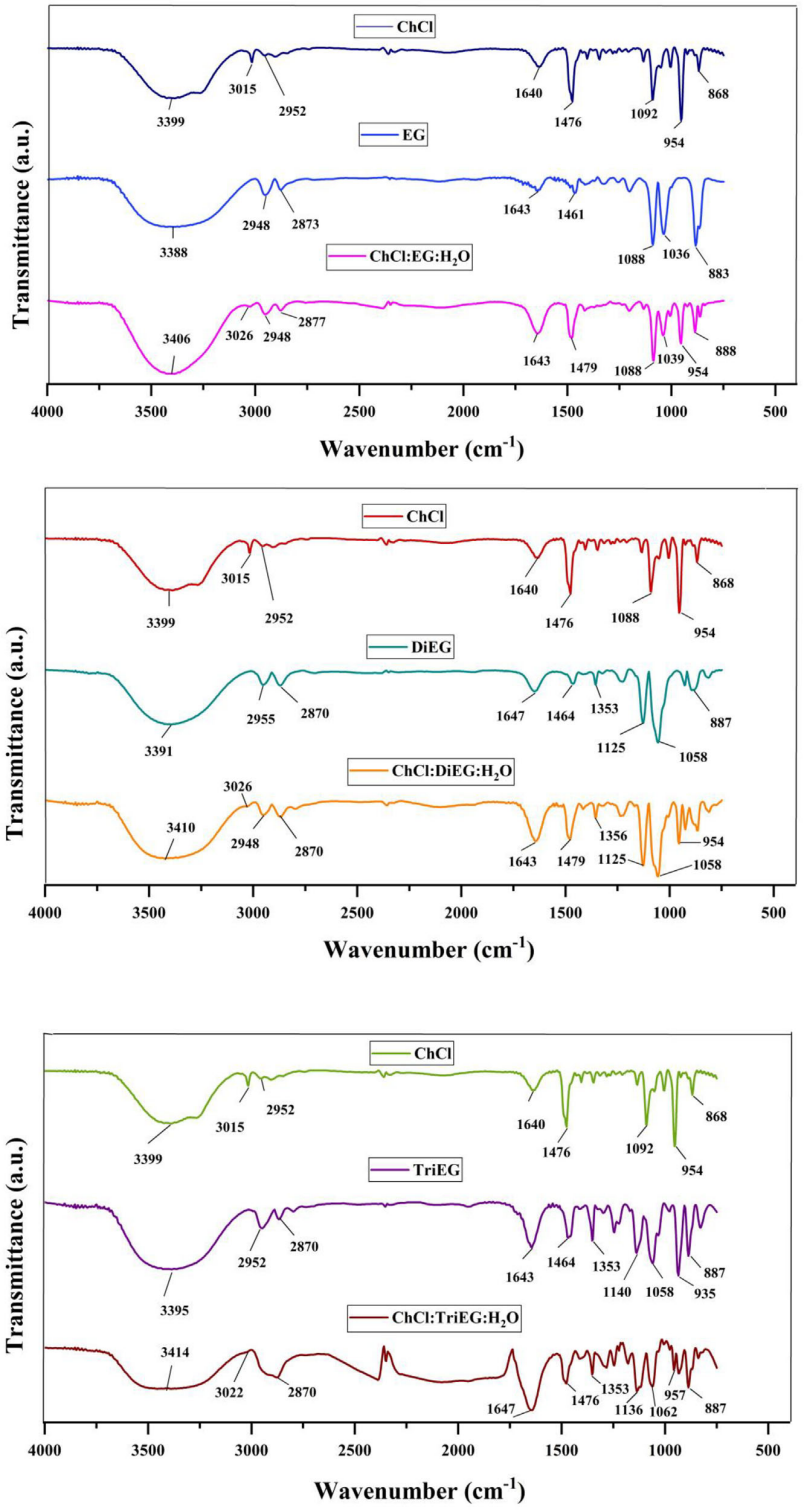

**Figure:**
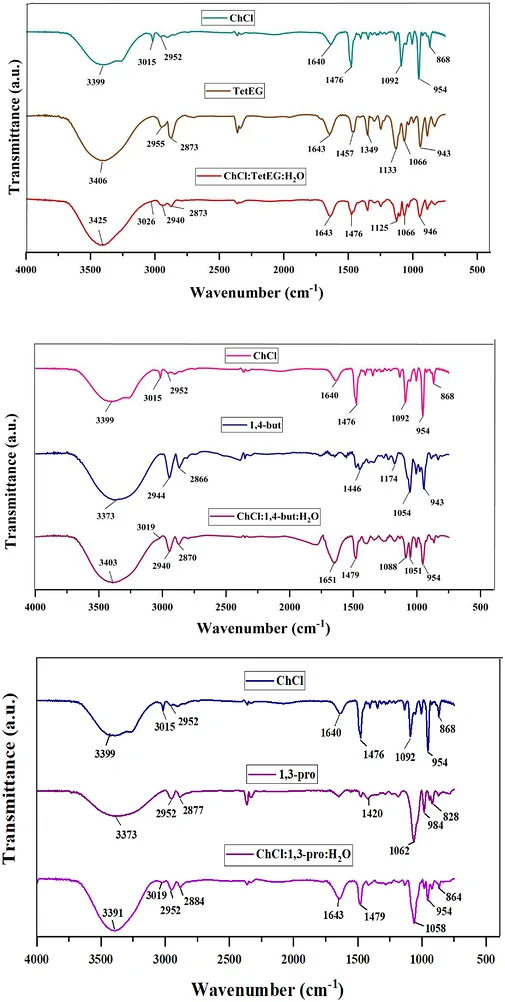

In addition, N─H bending vibrations were identified at 1640–1651 cm^−1^, whereas C─H bending modes were observed at approximately 1476–1479 cm^−1^. Vibrational bands corresponding to C─C and C─O stretching were located within the 1300–1000 cm^−1^ region. Furthermore, the absorption band near 954 cm^−1^ was assigned to C─N stretching vibrations, which are characteristic of ammonium‐containing structures [11, 18, 29, 30, 31].

A pronounced broadening of the O─H stretching band in the 3000–3500 cm^−1^ region was observed following DES formation, providing strong evidence for extensive hydrogen‐bonding interactions within the eutectic systems. In contrast to the sharp O─H stretching bands of the individual components, the broadened profiles in the DES spectra indicate the establishment of multiple hydrogen‐bonding interactions [32, 33].

The absorption band near 954 cm^−1^, attributed to C─N stretching of the ammonium group in ChCl, remained clearly visible in the DES spectra, confirming that the core ChCl was preserved after eutectic formation [28, 32].

### 3.2 MAE Optimization Results

The MAE process for *Vitex agnus‐castus* leaves was optimized using a solvent system composed of ChCl and TetEG, supplemented with 30% (v/v) water. The effects of the main extraction variables—microwave power, extraction time, and L/S ratio—on TAC were investigated using a BBED. A total of 17 experimental runs, including three replicated center points, were conducted, with TAC selected as the response variable. The BBED matrix is shown in Table 4. The experimental data were fitted to a second‐order polynomial model describing the relationship between TAC and the independent variables, as expressed in Equation (6).

(6) $$\begin{array}{ccc} & & \text{TAC}:+24.54+16.99A+23.00B-17.34C+16.52\text{AB}\\ & & \;-15.57\text{AC}-16.75\text{BC}+1.53A^{2}+8.38B^{2}+1.30C^{2} \end{array}$$

**TABLE 4:** Experimental Box–Behnken design (BBED) matrix showing the effects of microwave power (W), extraction time (s), and liquid‐to‐solid ratio (mL/g) on the total antioxidant capacity (TAC) of *Vitex agnus‐castus* leaf extracts obtained by MAE.

Run	A‐Power (Watt)	B‐Time (sec)	C‐L/S ratio (mL/g)	Response: TAC (mg TE/g sample)
1	250	60	20	15.100
2	200	60	40	7.550
3	250	90	40	24.537
4	300	90	20	74.559
5	200	90	60	11.325
6	300	60	40	11.325
7	300	120	40	94.379
8	300	90	60	11.325
9	250	120	20	90.604
10	250	90	40	24.538
11	250	60	60	11.325
12	200	120	40	24.536
13	250	90	40	24.535
14	250	90	40	24.538
15	250	90	40	24.538
16	200	90	20	12.269
17	250	120	60	19.819

In the developed regression model, factor A corresponds to microwave power (W), factor B represents extraction time (s), and factor C denotes the L/S ratio. The statistical significance, reliability, and predictive capability of the model were evaluated by ANOVA, as summarized in Table 5. The high F‐value (*F* = 157.10), together with a p‐value lower than 0.0001, confirms the strong statistical significance of the model, indicating that the observed effects are highly unlikely to be attributed to random error. In general, higher *F*‐values and lower *p*‐values indicate a greater contribution of the corresponding variables to the response. According to the ANOVA results, the linear terms (A, B, and C) describe the main effects of the variables, the quadratic terms (A^2^, B^2^, and C^2^) account for curvature effects, and the interaction terms (AB, AC, and BC) reflect the combined influence of variable pairs. At a 95% confidence level, all linear terms, all interaction terms, and the quadratic term B^2^ were found to be statistically significant.

**TABLE 5:** ANOVA results evaluating the statistical significance and adequacy of the quadratic regression model developed for optimizing MAE conditions of *Vitex agnus‐castus* leaf extracts using the BBED approach.

Source	Sum of Squares	df	Mean Square	*F*‐value	*p*‐value	
Model	12458.86	9	1384.32	157.10	< 0.0001	Significant
A‐Power (Watt)	2308.87	1	2308.87	262.02	< 0.0001	
B‐Time (s)	4233.75	1	4233.75	480.47	< 0.0001	
C‐L/S ratio (mL/g)	2406.03	1	2406.03	273.05	< 0.0001	
AB	1091.25	1	1091.25	123.84	< 0.0001	
AC	970.01	1	970.01	110.08	< 0.0001	
BC	1122.59	1	1122.59	127.40	< 0.0001	
A^2^	9.91	1	9.91	1.12	0.3242	
B^2^	295.43	1	295.43	33.53	0.0007	
C^2^	7.10	1	7.10	0.8056	0.3993	
Residual	61.68	7	8.81			
Lack of Fit	61.68	3	20.56			Significant
Pure Error	6.800E‐06	4	1.700E‐06			
Cor Total	12520.55	16				

The precision and robustness of the model were further supported by the fitting statistics presented in Table 6. The relatively low standard deviation (Std. Dev = 2.97) indicates that the experimental values are closely clustered around the predicted values, demonstrating good repeatability of the extraction process. Additionally, the low coefficient of variation (C.V.% = 9.96%) reflects minimal experimental variability and high reliability of the obtained results. The model showed excellent agreement with the experimental data, as evidenced by the high R^2^ (*R* ^2^ = 0.9951) and adjusted R^2^ value (adj *R* ^2^ = 0.9887), indicating that the model explains more than 99% of the variability in TAC. Moreover, the predicted R^2^ value of 0.9212 confirms the strong predictive capability of the model and demonstrates good agreement between the predicted and experimental responses. The adequate precision value of 36.31 further indicates a satisfactory signal‐to‐noise ratio, demonstrating that the selected variables adequately describe the MAE process. Although the lack‐of‐fit test was statistically significant, the model was considered appropriate for process optimization because of its excellent goodness‐of‐fit statistics and successful experimental validation under the predicted optimum conditions. The observed lack‐of‐fit may be attributed to the inherent biological variability of the plant material, experimental variability associated with the microwave‐assisted extraction process, and the limitations of the quadratic polynomial model in fully describing the complex nonlinear relationships among the extraction variables.

**TABLE 6:** Statistical parameters describing the goodness of fit and reliability of the ANOVA model.

Statistical parameters	
Std. Dev.	2.97
Mean	29.81
C.V.%	9.96
*R* ^2^	0.9951
Adj *R* ^2^	0.9887
Pre *R* ^2^	0.9212
Adeq. Precision	36.3246

The interactive effects of the extraction parameters on TAC are illustrated by the three‐dimensional response surface plots shown in Figure 3, which were generated based on the developed polynomial model. Each response surface plot depicts the effect of two independent variables on TAC while the remaining variable is held constant at its central level, allowing visualization of their combined influence on extraction performance. The curved nature of the response surfaces indicates the presence of significant interaction and quadratic effects among the extraction variables.

**FIGURE 2:**
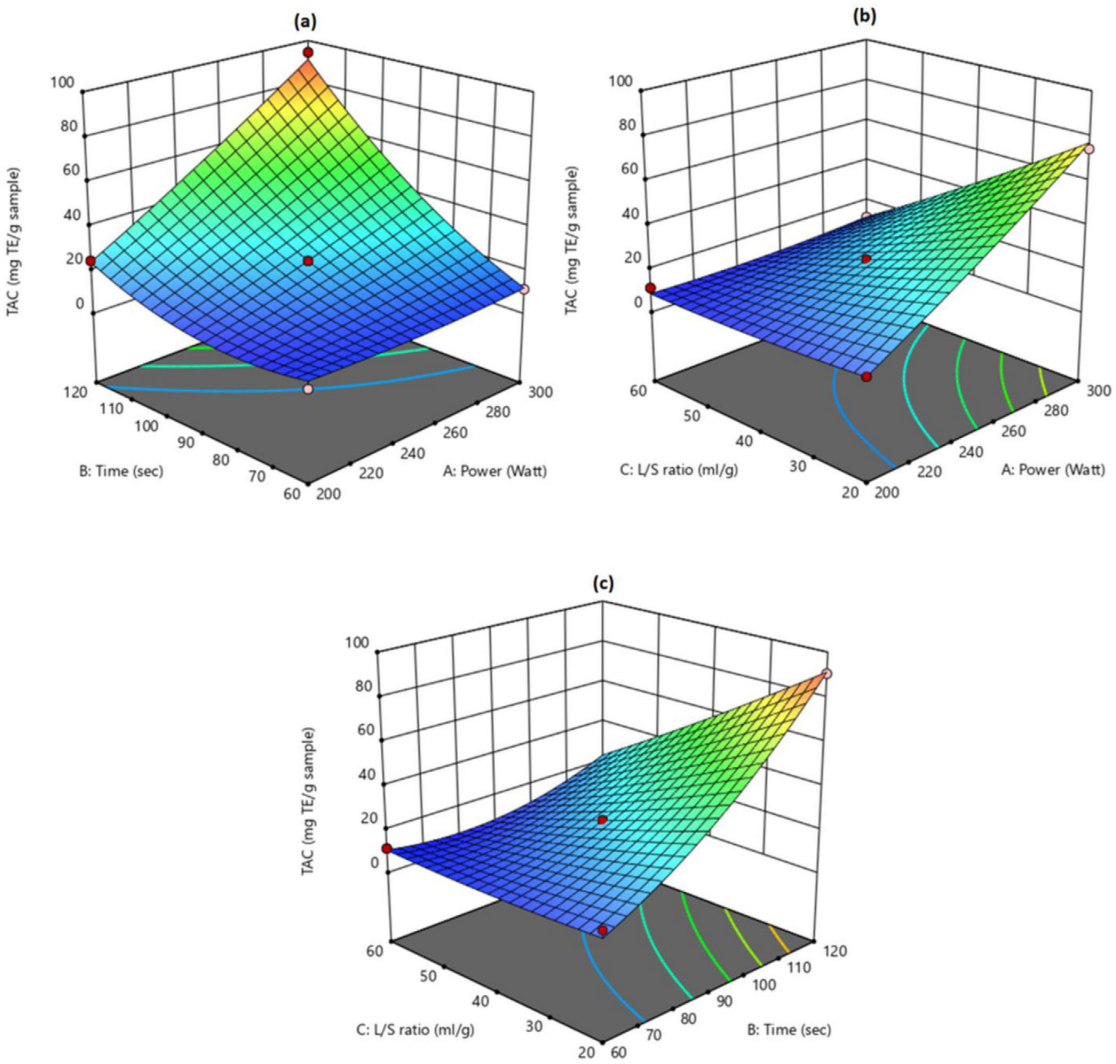
Three‐dimensional response surface plots illustrating the combined effects of (a) extraction time and microwave power, (b) liquid‐to‐solid ratio and microwave power, and (c) liquid‐to‐solid ratio and extraction time on the total antioxidant capacity (TAC) of *Vitex agnus‐castus* leaf extracts obtained by MAE.

The highest TAC was obtained at an extraction time of 120 s and an microwave power of 300 W, which can be attributed to sufficient energy input enabling effective disruption of plant cell walls without inducing thermal degradation (Figure 2a) [11, 21, 23, 33, 34]. Similarly, maximum TAC values were observed at a microwave power of 300 W combined with the lowest L/S ratio, likely due to increased energy density and reduced solvent dilution, which enhance mass transfer efficiency (Figure 2b) [11, 34]. Furthermore, the highest TAC was achieved at an extraction time of 120 s and an L/S ratio of 20, indicating that prolonged microwave extraction under more concentrated solvent conditions promotes the release of antioxidant compounds (Figure 2c) [11, 20, 25, 35, 36]. These response surface plots clearly demonstrate the combined influence of extraction parameters on TAC and confirm the suitability of RSM for optimizing the MAE process.

The optimal extraction conditions predicted by RSM were a microwave power of 293 W, an extraction time of 119 s, and an L/S ratio of 25.12. Under these conditions, the predicted TAC was 94.00 mg TE/g sample. To validate the developed model, the extraction experiment was performed three times under the predicted optimum conditions. The experimental TAC was 95.00 TE/g sample, corresponding to a percentage error of 1.06% compared with the predicted value. The close agreement between the predicted and experimental TAC values confirms the validity, reliability, and predictive capability of the developed RSM model.

### 3.3 UAE Optimization Results

The UAE process for *Vitex agnus‐castus* leaves was optimized using a solvent system composed of ChCl and TriEG, supplemented with 30% (v/v) water. The principal extraction variables—ultrasonic power, extraction time, and L/S ratio—were systematically optimized using a BBED. A total of 17 experimental runs, including three replicated center points to ensure model reliability, were performed, with TAC selected as the response variable (Table 7). The experimental data obtained from the design were subjected to regression analysis and fitted to a second‐order polynomial model describing the quantitative relationship between TAC and the selected independent variables, as presented below.

(7) $$\begin{array}{ccc} & & \text{TAC}:+117.40+12.76A+3.99B-7.50C-8.93\text{AB}+7.02\text{AC}\\ & & +0.9573\text{BC}\text{--}8.45A^{2}\text{--}13.24B^{2}\text{--}4.95C^{2} \end{array}$$

**TABLE 7:** Experimental Box–Behnken design matrix presenting the effects of ultrasonic power (W), extraction time (min), and liquid‐to‐solid ratio (mL/g) on the total antioxidant capacity (TAC) of *Vitex agnus‐castus* leaf extracts obtained by UAE.

Run	A‐Power (W)	B‐Time (min)	C‐L/S ratio (mL/g)	Response:TAC (mg TE/g sample)
1	300	20	100	89.328
2	500	10	60	107.193
3	700	30	80	112.298
4	500	20	80	117.403
5	500	20	80	117.401
6	500	20	80	117.404
7	500	30	60	111.022
8	300	10	80	61.253
9	500	20	80	117.403
10	300	20	60	111.022
11	500	10	100	82.947
12	500	30	100	114.850
13	700	20	100	108.469
14	700	20	60	107.193
15	500	20	80	117.402
16	300	30	80	112.298
17	700	10	80	96.984

In the regression model, factor A denotes ultrasonic power (W), factor B corresponds to extraction time (min), and factor C represents the L/S ratio (ml/g). The robustness and predictive reliability of the model were evaluated using ANOVA, with the results summarized in Table 8. Model significance was assessed based on *F*‐ and *p*‐values, where the high *F*‐value (7.92), together with a p‐value below 0.05, clearly demonstrates strong statistical significance, indicating that the likelihood of the observed effects arising from random variation is minimal [37, 38].

**TABLE 8:** ANOVA results assessing the statistical significance, reliability, and predictive performance of the quadratic regression model developed for UAE optimization using the BBED approach.

Source	Sum of Squares	df	Mean Square	*F*‐value	*p*‐value	
Model	3651.45	9	405.72	7.92	0.0062	Significant
A‐Power (Watt)	127.22	1	127.22	2.48	0.1591	
B‐Time (min)	1302.82	1	1302.82	25.43	0.0015	
C‐L/S ratio (ml/g)	449.66	1	449.66	8.78	0.0210	
AB	319.18	1	319.18	6.23	0.0412	
AC	3.67	1	3.67	0.0715	0.7968	
BC	197.04	1	197.04	3.85	0.0907	
A^2^	738.07	1	738.07	14.40	0.0068	
B^2^	300.97	1	300.97	5.87	0.0459	
C^2^	102.96	1	102.96	2.01	0.1993	
Residual	358.67	7	51.24			
Lack of Fit	358.67	3	119.56	9.197E+07	< 0.0001	Significant
Pure Error	5.200E‐06	4	1.300E‐06			
Cor Total	4010.12	16				

The reliability and consistency of the developed model were further supported by the fitting statistics presented in Table 9. The relatively low Std. Dev. value (Std. Dev. = 7.16) indicates that the experimental results are closely distributed around the predicted mean values, confirming good repeatability of the extraction process. Moreover, the low C.V.% (C.V.% = 6.83 %) reflects minimal dispersion among the experimental data and high methodological consistency. The adequacy of the model is reinforced by a *R* ^2^ of 0.9605 and an adj *R* ^2^ of 0.9171, demonstrating that the model explains a substantial proportion of the variability observed in TAC. In addition, the adequate precision value of 8.6295 indicates a satisfactory signal‐to‐noise ratio, confirming that the selected variables effectively describe the behavior of the UAE process. Although the lack‐of‐fit test was statistically significant, the model was considered suitable for optimization because it exhibited good predictive performance and was successfully validated under the predicted optimum conditions. The observed lack‐of‐fit may be associated with the inherent variability of the plant matrix, experimental variability during ultrasound‐assisted extraction, and the limited ability of the quadratic polynomial model to fully capture the complex interactions among the extraction variables over the investigated experimental range.

**TABLE 9:** Statistical adequacy parameters and goodness‐of‐fit indicators derived from ANOVA for evaluating the reliability and predictive capability of the UAE optimization model.

Statistical parameters	
Std. Dev.	7.16
Mean	104.87
C.V. %	6.83
R^2^	0.9605
Adjusted R^2^	0.9171
Adeq Precision	8.6295

**FIGURE 3:**
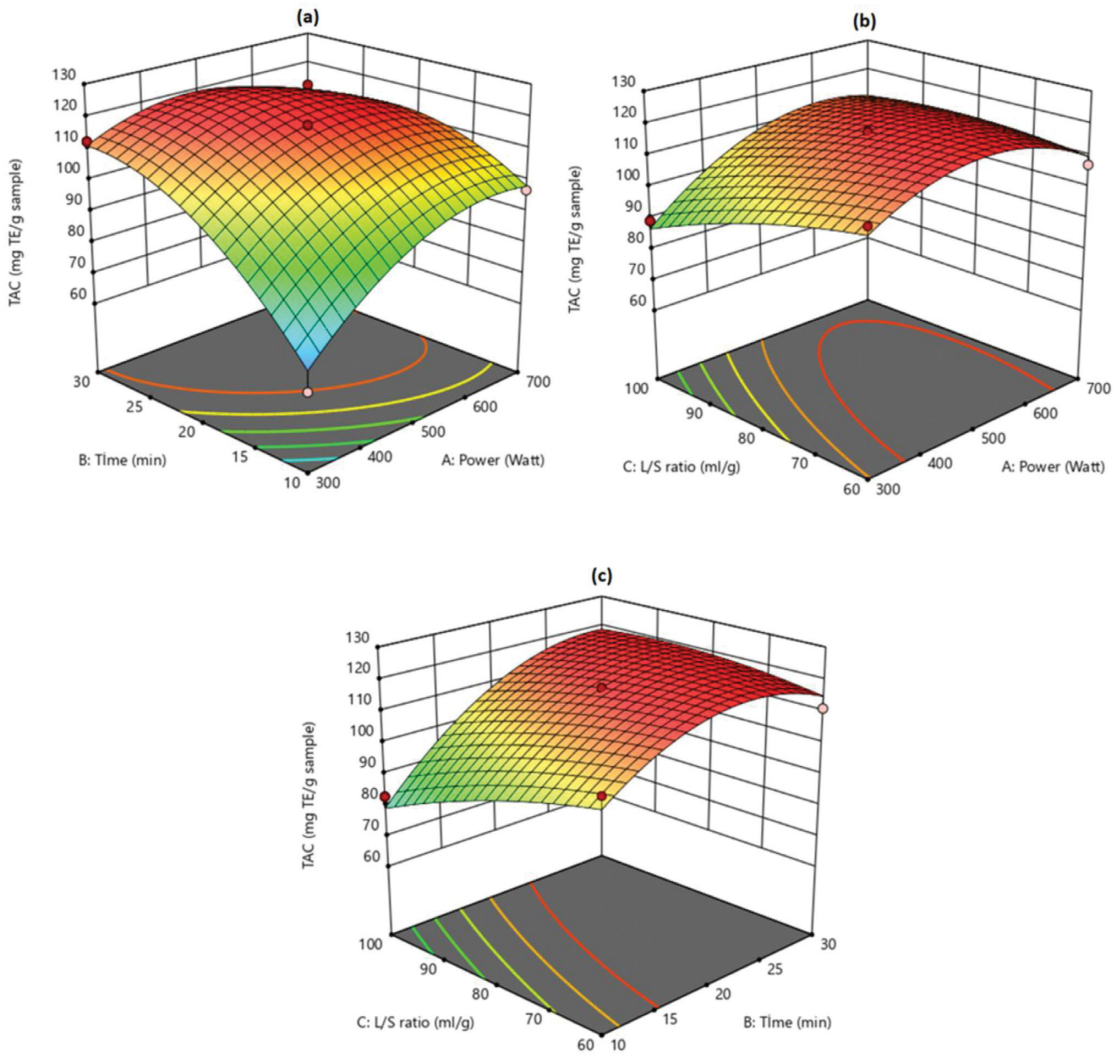
Three‐dimensional response surface plots showing the combined effects of (a) extraction time and ultrasonic power, (b) liquid‐to‐solid ratio and ultrasonic power, and (c) liquid‐to‐solid ratio and extraction time on the total antioxidant capacity (TAC) of *Vitex agnus‐castus* leaf extracts obtained by UAE.

The interactive effects of the extraction parameters on TAC are illustrated by the three‐dimensional response surface plots shown in Figure 3, which were generated using the developed polynomial model. Each response surface plot depicts the influence of two independent variables on TAC while the remaining variable is maintained at its central level, thereby enabling visualization of their combined effects on extraction performance.

As shown in Figure 3a, increasing ultrasonic power and extraction time enhanced TAC values up to approximately 500 W. This trend can be attributed to increased cavitation intensity and internal cell pressure, which promote cell wall disruption and facilitate the release of antioxidant compounds. However, a decrease in TAC was observed beyond 500 W with prolonged extraction time, suggesting that excessive energy input may lead to thermal or oxidative degradation of antioxidants. As illustrated in Figure 3b, the highest TAC values were obtained under intermediate conditions, particularly at an ultrasonic power of 500 W and an L/S ratio of 70. This behavior likely reflects an optimal balance between energy intensity and solvent availability, which enhances mass transfer without causing excessive dilution or compound degradation. Additionally, relatively high TAC values were observed in Figure 3c at an L/S ratio of 80 combined with an extraction time of 30 min, possibly due to sufficient solvent penetration and controlled ultrasonic exposure [11, 20, 25, 34, 35, 36].

Based on RSM, the optimal extraction conditions were predicted to be an ultrasonic power of 501.96 W, an extraction time of 23.27 min, and an L/S ratio of 70.08, corresponding to a predicted TAC value of 117.63 mg TE/g sample. To validate the developed model, the extraction experiment was performed three times under the predicted optimum conditions. The experimental TAC was 123.76 mg TE/g sample, corresponding to a percentage error of 5.21% compared with the predicted value. The close agreement between the predicted and experimental TAC values confirms the validity, reliability, and predictive capability of the developed RSM model.

The differences observed between MAE and UAE can be explained by their fundamentally different extraction mechanisms. MAE is based on the rapid heating effect generated by microwave energy, which increases internal cell pressure and promotes the rupture of plant cell walls, thereby facilitating the release of intracellular phenolic compounds. In contrast, UAE operates through acoustic cavitation, where the formation and collapse of microbubbles generate localized shear forces that enhance solvent penetration and mass transfer. Due to these mechanistic differences, MAE generally enables faster and more efficient extraction through direct volumetric heating, whereas UAE provides milder extraction conditions that may better preserve thermolabile bioactive compounds.

### 3.4 Antioxidant Activity Results

Antioxidant activity parameters obtained under optimized MAE and UAE conditions using DES and 70% EtOH were comparatively presented and discussed in Table 10. In this study, TAC values obtained from different parts of *Vitex agnus‐castus* using DES in both MAE and UAE were generally comparable to those obtained with 70% EtOH (Table 10), although slightly higher values were observed for most DES‐based extracts, with the exception of leaf extracts produced by MAE using DES. All subsequent analyses were performed under the extraction conditions optimized according to TAC values. The similar extraction efficiencies observed between DES and EtOH suggest that both solvents are capable of effectively solubilizing antioxidant compounds from *Vitex agnus‐castus*. The extraction performance of DES may be partly attributed to hydrogen‐bonding interactions between the hydroxyl groups of phenolic compounds and the anionic species of the ChCl‐based DES system, as well as to synergistic interactions between DES components and bioactive molecules, which can contribute to improved extraction efficiency while minimizing the degradation of labile phenolics and flavonoids and thereby supporting extract stability and quality [17, 39]. In addition, the physicochemical properties of DESs, including polarity, viscosity, and hydrogen‐bonding capacity, are known to strongly influence mass transfer efficiency and the solubilization of phenolic compounds during extraction. The presence of water in the DES formulations may also reduce viscosity and enhance solvent penetration into plant matrices, thereby facilitating the release of antioxidant constituents.

**TABLE 10:** Comparison of antioxidant activity parameters of *Vitex agnus‐castus* extracts obtained under optimized MAE and UAE conditions using DES and 70% EtOH as extraction solvents.

	TAC‐MAE (mg TE/g‐DS)	TAC‐MAE (mg TE/g‐DS)	DPPH‐MAE (mg TE/g‐DS)	DPPH‐MAE (mg TE/g‐DS)	ABTS‐MAE (mg TE/g‐DS)	ABTS‐MAE (mg TE/g‐DS)	TAC‐UAE (mg TE/g‐DS)	TAC‐ UAE (mg TE/g‐DS)	DPPH‐ UAE (mg TE/g‐DS)	DPPH‐ UAE (mg TE/g‐DS)	ABTS‐ UAE (mg TE/g‐DS)	ABTS‐ UAE (mg TE/g‐DS)
Plant	DES	%70 EtOH	DES	%70 EtOH	DES	%70 EtOH	DES	%70 EtOH	DES	%70 EtOH	DES	%70 EtOH
Leaves	94.83	230.20	100.60	101.92	70.28	77.06	123.76	88.00	59.43	49.96	32.71	25.24
Flowers	119.69	76.38	74.53	39.10	100.15	39.74	74.27	60.05	40.79	32.51	22.54	22.60
Seeds	39.52	21.49	2.13	8.58	28.65	13.01	35.18	14.81	96.60	4.97	5.08	5.21

The comparatively higher TAC values relative to those obtained from ABTS and DPPH assays can be attributed to the broader scope of the TAC method, which reflects the overall antioxidant potential of diverse compounds, many of which are not effective radical scavengers in ABTS or DPPH systems. In addition to phenolic constituents, various non‐phenolic antioxidants—such as certain vitamins, amino acids, and inorganic components—may substantially contribute to TAC measurements [40]. In particular, the ABTS radical cation demonstrates limited reactivity toward lipophilic antioxidants and is characterized by slower reaction kinetics, which may result in underestimated antioxidant activity. Moreover, because ABTS^+^ is a nitrogen‐centered radical, its restricted interaction with polymeric phenolic structures further limits reaction efficiency and influences the final assay results [41].

The enhanced extraction efficiency of DESs may be associated with their strong hydrogen‐bonding capacity and adjustable polarity, which facilitate interactions with phenolic compounds and improve their solubilization. In addition, the distinct extraction mechanisms of MAE and UAE contribute to differences in antioxidant recovery, where MAE promotes rapid internal heating and cell wall disruption, while UAE enhances mass transfer through acoustic cavitation effects.

Overall, the findings indicate that DES and 70% EtOH exhibited largely comparable efficiencies in the extraction of antioxidant compounds from *Vitex agnus‐castus*. The observed similarities in antioxidant parameters suggest that the phytochemical profile and antioxidant potential of *Vitex agnus‐castus* are strongly dependent on intrinsic factors such as plant part, genotype, cultivation conditions, storage, and the applied extraction technique rather than solely on solvent type [42, 43]. Although DES offers advantages in terms of sustainability and green chemistry principles, the comparable performance of EtOH indicates that conventional solvents may still provide effective extraction under appropriate conditions. Nevertheless, considering its low toxicity, tunable properties, and potential to improve extraction selectivity and bioavailability, DES remains a promising alternative solvent system for the recovery of antioxidants from *Vitex agnus‐castus* and other medicinal plant materials.

Overall, the antioxidant activity results indicate that the extraction efficiency was strongly influenced by the combined effects of the solvent system and extraction technique. While DES‐based extracts showed enhanced recovery under several conditions, 70% EtOH exhibited superior performance in certain MAE applications, particularly for leaf extracts. These findings suggest that the effectiveness of DES, MAE, and UAE is highly dependent on the interactions between solvent properties, extraction mechanisms, and the specific phytochemical composition of the plant material. The results also indicate that DES should not be considered universally superior to conventional solvents in terms of extraction efficiency. In the present study, 70% ethanol produced substantially higher TAC values than DES for leaf extracts obtained by MAE, which may be attributed to the more efficient interaction of aqueous ethanol with microwave energy. Owing to its favorable dielectric properties, 70% ethanol absorbs microwave irradiation more efficiently, resulting in enhanced volumetric heating, improved cell wall disruption, and increased mass transfer. In contrast, the higher viscosity and different dielectric characteristics of DES may reduce microwave energy transfer efficiency under the investigated conditions. Nevertheless, the primary advantage of DES lies in its environmental sustainability, low toxicity, biodegradability, and tunable physicochemical properties rather than consistently higher extraction efficiency. Therefore, the selection of an extraction solvent should be based not only on extraction yield but also on environmental and sustainability considerations, depending on the intended application.

### 3.5 HPLC Results

The most pronounced extraction performance was achieved using leaf samples subjected to MAE with 70% EtOH. This specific solvent–extraction technique combination enabled the effective solubilization and recovery of caffeic acid and syringic acid, which were not detected in extracts obtained using other solvent or extraction method combinations (Table 11). Moreover, the highest rutin concentration was also obtained under these conditions, indicating that MAE combined with 70% EtOH is particularly effective for the extraction of certain phenolic acids and flavonoids from the leaf matrix. This enhanced performance may be attributed to the efficient dielectric heating provided by microwave irradiation, which facilitates cell wall disruption and improves solvent penetration into plant tissues.

**TABLE 11:** Phenolic and flavonoid compounds detected in mg/g sample in different parts of *Vitex agnus‐castus*.

		Caffeic acid	Syringic acid	*p*‐coumaric acid	Ferulic acid	Rutin	Myricetin	Quercetin	Kaempferol
Leaves‐ MAE	DES	—	—	15.72	—	9.28	—	—	—
Flowers‐MAE	DES	—	—	15.16	—	3.26	—	—	—
Seeds‐MAE	DES	—	—	—	—	—	—	—	—
Leaves‐MAE	%70 EtOH	3.94	8.72	22.72	—	39.09	2.30	—	—
Flowers‐MAE	%70 EtOH	—	—	6.42	—	11.32	2.84	—	—
Seeds‐MAE	%70 EtOH	—	—	—	—	—	—	—	—
Leaves‐ UAE	DES	—	—	2.00	—	20.94	—	—	—
Flowers‐UAE	DES	—	—	6.40	—	—	—	—	—
Seeds‐UAE	DES	—	—	—	—	—	—	—	—
Leaves‐ UAE	%70 EtOH	—	—		—	—	—	—	—
Flowers‐UAE	%70 EtOH	—	—	—	—	—	—	—	—
Seeds‐UAE	%70 EtOH	—	—	—	—	—	—	—	—

In contrast, phenolic and flavonoid compounds were not detected in seed samples within the investigated concentration range. This observation may be explained by the compositional characteristics of *Vitex agnus‐castus* seeds, which are known to contain a high proportion of lipophilic constituents, particularly oils, rather than polar antioxidant compounds. The predominance of lipid components in the seed matrix may limit the extractability or detectability of phenolic and flavonoid compounds under the applied extraction conditions.

Overall, MAE generally resulted in higher extraction yields and antioxidant‐related parameters compared to UAE. For example, MAE using 70% EtOH for leaf extracts yielded higher amounts of p‐coumaric acid (22.72 vs. 0 mg/g‐DS), rutin (39.09 vs. 0 mg/g‐DS), and myricetin (2.30 vs. 0 mg/g‐DS) compared to UAE. Similarly, DES‐based MAE extracts of leaves contained higher p‐coumaric acid levels (15.72 vs. 2.00 mg/g‐DS) than DES‐based UAE extracts. This trend suggests a more synergistic interaction between microwave energy and the phenolic constituents of *Vitex agnus‐castus*, potentially due to enhanced mass transfer, rapid internal heating, and improved disruption of plant cellular structures. The selective detection of phenolic compounds in specific extracts further indicates that both solvent composition and extraction technique strongly influenced extraction selectivity. The superior recovery of caffeic acid, syringic acid, rutin, and myricetin in MAE extracts prepared with 70% EtOH is likely associated with the favorable polarity of aqueous ethanol and its efficient interaction with microwave energy, which enhanced cell wall disruption and facilitated the release of these compounds. In contrast, the absence of detectable phenolic compounds in several UAE extracts may indicate that their concentrations were below the detection limit of the HPLC method or that the selected extraction conditions were less effective for recovering the targeted analytes. Furthermore, the HPLC analysis was limited to selected phenolic acids and flavonoids; therefore, other antioxidant constituents not included in the analytical method may also have contributed to the overall antioxidant activity. This interpretation is supported by the antioxidant assays, where several extracts exhibited considerable TAC, DPPH, and ABTS activities despite relatively low concentrations of the quantified phenolic compounds, suggesting that unidentified phenolic and non‐phenolic antioxidants also contributed to the observed antioxidant capacity. These findings highlight the importance of selecting appropriate solvent–method combinations tailored to both plant part and target compounds to maximize extraction efficiency. The HPLC results further support the antioxidant activity findings. In particular, extracts containing higher concentrations of phenolic compounds, especially rutin, *p‐*coumaric acid, caffeic acid, and syringic acid, generally exhibited higher TAC values, indicating that these compounds contributed substantially to the overall antioxidant capacity. However, the relationship between phenolic content and radical scavenging activity was not always proportional. Several extracts exhibited relatively high TAC despite moderate DPPH and ABTS activities, suggesting that antioxidant capacity was also influenced by other phenolic and non‐phenolic constituents that were not quantified in the targeted HPLC analysis. This finding highlights the complexity of the antioxidant profile of *Vitex agnus‐castus* extracts and indicates that different antioxidant assays reflect different mechanisms of antioxidant action.

These findings are consistent with previous studies on *Vitex agnus‐castus*, which have primarily relied on conventional solvent extraction methods. For example, Latoui et al. reported a maximum TPC of 31.5 mg GAE g^−1^ dry biomass and the highest DPPH antiradical activity only after prolonged extraction times of 180–360 min using methanolic extraction [44]. Similarly, Drioua et al. evaluated aqueous, methanolic, and ethanolic maceration extracts and reported antioxidant activities using conventional extraction techniques [45]. In contrast, the present study demonstrates that DES‐based MAE and UAE can efficiently recover bioactive compounds using substantially shorter extraction times (119 s for MAE and 23.27 min for UAE). Moreover, unlike previous reports, this study integrates DES physicochemical characterization, response surface optimization, comparison with conventional 70% ethanol extraction, HPLC profiling of individual phenolic and flavonoid compounds, and antibacterial evaluation, thereby providing the first comprehensive assessment of DES‐assisted MAE and UAE for *Vitex agnus‐castus*.

### 3.6 Microbial Activity Results

The antibacterial activity of extracts obtained from different parts (leaves, flowers, and seeds) of *Vitex agnus‐castus* using different extraction methods (UAE and MAE) was evaluated against a range of pathogenic bacteria, including MRSA (LY 1999 0053), *Klebsiella pneumoniae* ATCC 33495, *Streptococcus pneumoniae* ATCC 49619, and *Pseudomonas aeruginosa* ATCC 27853 (Table 12).

**TABLE 12:** Antibacterial activity of ethanolic extracts of *Vitex agnus‐castus* obtained using different extraction methods.

Bacteria	Leaves UAE	Leaves MAE	Flowers UAE	Flowers MAE	Seeds UAE	Seeds MAE	Ciprofloxacin
MRSA	MIC: 25 µg/mL Zd: 14 mm	MIC: 50 µg/mL Zd: 15 mm	MIC: 25 µg/mL Zd: 11 mm	MIC: 50 µg/mL Zd: 12 mm	MIC: 25 µg/mL Zd: 16 mm	MIC: 50 µg/mL Zd: 13 mm	MIC: 2 µg/mL Zd: 22 mm
*S. pneumoniae*	MIC: 6.75 µg/mL Zd: 10 mm	MIC: 12.5 µg/mL Zd: 11 mm	MIC: 12.5 µg/mL Zd: 11 mm	MIC: 25 µg/mL Zd: 12 mm	MIC: 6.75 µg/mL Zd: 14 mm	MIC: 25 µg/mL Zd: 12 mm	MIC: 0.5 µg/mL Zd: 24 mm
*K.pneumoniae*	MIC: 100 µg/mL Zd: 14 mm	MIC: 200 µg/mL Zd: 11 mm	MIC: 200 µg/mL Zd: 11 mm	MIC: 400 µg/mL Zd: 12 mm	MIC: 100 µg/mL Zd: 13 mm	MIC: 200 µg/mL Zd: 12 mm	MIC: 1 µg/mL Zd: 26 mm
*P. aeruginosa*	MIC: 200 µg/mL Zd: 9 mm	MIC: 400 µg/mL Zd: 14 mm	MIC: 400 µg/mL Zd: 12 mm	MIC: 400 µg/mL Zd: 10 mm	MIC: 200 µg/mL Zd: 14 mm	MIC: 400 µg/mL Zd: 11 mm	MIC: 4 µg/mL Zd: 20 mm

^a^ Zone diameter: Zd.

The MIC values of the leaf and flower UAE extracts against MRSA strains were 25 µg/mL, with inhibition zone diameters of 14 mm and 11 mm, respectively. The seed UAE extract also exhibited an MIC of 25 µg/mL and a zone diameter of 16 mm. In subsequent experiments, the MIC values increased to 50 µg/mL, while the inhibition zones ranged between 12 and 15 mm. The positive control, ciprofloxacin, showed the highest antibacterial activity, with an MIC of 2 µg/mL and a zone diameter of 22 mm.

The MIC values for the leaf and seed UAE extracts were found to be the lowest at 6.75 µg/mL, with zone diameters of 10 mm and 14 mm, respectively. The MIC of the flower UAE extract was determined to be 12.5 µg/mL, accompanied by a zone diameter of 11 mm. For the MAE extracts, MIC values ranged from 12.5 to 25 µg/mL, with inhibition zones measuring between 11‐ and 12‐mm. Ciprofloxacin again exhibited the strongest inhibitory effect, demonstrating an MIC of 0.5 µg/mL and a zone diameter of 24 mm.

Against *Klebsiella pneumoniae* strains, leaf and seed extracts produced inhibition zones of 14 mm and 13 mm, respectively, corresponding to an MIC value of 100 µg/mL. The MIC of the UAE extract was determined to be 200 µg/mL, with a recorded zone diameter of 11 mm. The MIC values of the MAE extracts ranged from 200 to 400 µg/mL, with inhibition zone diameters between 11‐ and 12‐mm. Ciprofloxacin demonstrated the highest antibacterial activity against this bacterium, with an MIC of 1 µg/mL and a zone diameter of 26 mm.

For *Pseudomonas aeruginosa*, the MIC values of the leaf and seed UAE extracts were 200 µg/mL, with zone diameters of 9 mm and 14 mm, respectively. The flower UAE extract exhibited an MIC of 400 µg/mL and a zone diameter of 12 mm. Similarly, the MAE extracts showed MIC values of 400 µg/mL, with inhibition zones ranging from 10 to 14 mm. The positive control, ciprofloxacin, displayed significantly greater antibacterial activity than the plant extracts, with an MIC of 4 µg/mL and a zone diameter of 20 mm.

The results indicate that the extracts of *Vitex agnus‐castus* exhibited antibacterial activity that varied according to both the extraction method and the plant part used. Overall, UAE‐derived extracts generally demonstrated lower MIC values and slightly larger inhibition zones compared to MAE extracts under several conditions. Among the plant parts, leaf extracts showed the highest antibacterial activity, followed by seed and flower extracts. The most pronounced activity was observed against MRSA strains, whereas *Klebsiella pneumoniae* and *Pseudomonas aeruginosa* exhibited higher resistance. However, the antibacterial effects of all plant extracts remained lower than those of the standard antibiotic ciprofloxacin. Therefore, the antibacterial efficiency of *Vitex agnus‐castus* extracts appears to depend on the interaction between extraction technique and plant matrix rather than indicating the absolute superiority of one extraction method.

Although the antibacterial activities of the extracts were lower than those of ciprofloxacin, both MIC values and inhibition zone diameters consistently demonstrated measurable antibacterial effects against the tested microorganisms, as presented in Table 13. The combined evaluation of MIC and agar well diffusion results provided complementary information regarding bacterial growth inhibition and extract diffusion behavior. Nevertheless, the observed antibacterial activity should be interpreted within the limitations of the applied in vitro methods.

**TABLE 13:** Antibacterial activity of DES extracts of *Vitex agnus‐castus* obtained using different extraction methods.

Bacteria	Leaves UAE	Leaves MAE	Flowers UAE	Flowers MAE	Seeds UAE	Seeds MAE	Ciprofloxacin
MRSA	MIC: 50 µg/mL Zd: 10 mm	MIC: 100 µg/mL Zd: 11 mm	MIC: 100 µg/mL Zd: 9 mm	MIC: 200 µg/mL Zd: 10 mm	MIC: 50 µg/mL Zd: 13 mm	MIC: 100 µg/mL Zd: 11 mm	MIC: 2 µg/mL Zd: 22 mm
*S. pneumoniae*	MIC: 12.5 µg/mL Zd: 8 mm	MIC: 25 µg/mL Zd: 10 mm	MIC: 25 µg/mL Zd: 11 mm	MIC: 50 µg/mL Zd: 11 mm	MIC: 12.5 µg/mL Zd: 12 mm	MIC: 50 µg/mL Zd: 11 mm	MIC: 0.5 µg/mL Zd: 24 mm
*K.pneumoniae*	MIC: 200 µg/mL Zd: 11 mm	MIC: 400 µg/mL Zd: 9 mm	MIC: 400 µg/mL Zd: 8 mm	MIC: >400 µg/mL Zd: 9 mm	MIC: 200 µg/mL Zd: 11 mm	MIC: 400 µg/mL Zd: 8 mm	MIC: 1 µg/mL Zd: 26 mm
*P. aeruginosa*	MIC: 400 µg/mL Zd: 9 mm	MIC: >400 µg/mL Zd: 10 mm	MIC: >400 µg/mL Zd: 9 mm	MIC: >400 µg/mL Zd: 8 mm	MIC: 400 µg/mL Zd: 9 mm	MIC: >400 µg/mL Zd: 7 mm	MIC: 4 µg/mL Zd: 20 mm

^a^ Zone diameter: Zd.

With respect to MRSA strains, leaf and seed UAE extracts produced inhibition zone diameters of 10 mm and 13 mm, respectively, with MIC values of 50 µg/mL. In contrast, the MIC values of the MAE‐derived leaf, flower, and seed extracts ranged from 100 to 200 µg/mL, with inhibition zones measuring between 9 and 11 mm. As shown in Table 12, the UAE extract exhibited reduced antibacterial activity, characterized by an MIC of 100 µg/mL and a zone diameter of 9 mm.

For *Streptococcus pneumoniae*, the MIC values of the leaf and seed UAE extracts were 12.5 µg/mL, accompanied by inhibition zone diameters of 8 mm and 12 mm, respectively. The flower UAE extract showed an MIC of 25 µg/mL and a zone diameter of 11 mm. The MAE extracts exhibited MIC values ranging from 25 to 50 µg/mL, with inhibition zones between 10 and 11 mm.

Against *Klebsiella pneumoniae* strains, the MIC values of the leaf and seed UAE extracts were 200 µg/mL, with inhibition zones of 11 mm for both extracts. The flower UAE extract demonstrated a higher MIC value of 400 µg/mL and a mean inhibition zone diameter of 8 mm. In comparison, the MAE extracts showed MIC values exceeding 400 µg/mL, with inhibition zones ranging from 8 to 9 mm.

For *Pseudomonas aeruginosa*, the MIC values of the leaf and seed UAE extracts were 400 µg/mL, with inhibition zones of 9 mm. The flower UAE extract exhibited an MIC value greater than 400 µg/mL, with a zone diameter of 9 mm. Similarly, the MAE extracts demonstrated MIC values exceeding 400 µg/mL, with inhibition zones ranging from 7 to 10 mm. The positive control, ciprofloxacin, displayed significantly higher antibacterial activity than the plant extracts, with an MIC of 4 µg/mL and an inhibition zone diameter of 20 mm.

During the evaluation of the antibacterial activity of *Vitex agnus‐castus* extracts, *Streptococcus pneumoniae* was identified as the most sensitive bacterium among those tested. UAE–ethanol extracts obtained from the leaves and seeds exhibited notably low MIC values (6.75 µg/mL) and relatively large inhibition zones (10–14 mm). Although their activity was lower than that of the positive control, ciprofloxacin (MIC: 0.5 µg/mL; zone diameter: 24 mm), these extracts still demonstrated measurable antibacterial activity under the applied in vitro conditions. In contrast, flower extracts showed comparatively weaker activity against this bacterium, as reflected by higher MIC values ranging from 12.5 to 25 µg/mL.

Moderate antibacterial activity was observed against MRSA, a Gram‐positive strain. UAE‐derived leaf and seed extracts exhibited appreciable inhibition (MIC: 25 µg/mL; zone diameters: 14–16 mm), whereas extracts obtained using the MAE method showed reduced activity (MIC: 50 µg/mL; zone diameters: 12–15 mm). This reduction suggests that UAE may be more effective than MAE in preserving phenolic and volatile bioactive compounds.

In contrast, Gram‐negative bacteria displayed a distinct resistance profile. *Klebsiella pneumoniae* and *Pseudomonas aeruginosa* exhibited limited susceptibility to the extracts, with relatively high MIC values ranging from 100 to 400 µg/mL and smaller inhibition zones (9–14 mm). This observation supports the hypothesis that structural and functional defense mechanisms of Gram‐negative bacteria, such as the outer membrane barrier and efflux pump systems, restrict the antibacterial efficacy of plant‐derived compounds.

Among the different plant parts, seed extracts demonstrated the highest antibacterial activity, followed by leaf extracts, which showed moderate to high efficacy, particularly against Gram‐positive bacteria. Flower extracts consistently exhibited the weakest activity against both bacterial groups. These differences indicate that variations in the distribution and concentration of essential oils and phenolic constituents among plant parts play a critical role in determining antibacterial potency.

The choice of extraction solvent was also found to significantly influence antibacterial outcomes. DES enhanced the extraction efficiency of bioactive compounds by improving solubility and mass transfer, resulting in lower MIC values and larger inhibition zones. This highlights the potential of DES as green and effective alternatives to conventional solvents for the extraction of antimicrobial agents from medicinal plants.

Regarding energy‐assisted extraction techniques, UAE was shown to be particularly effective due to its cavitation‐induced disruption of plant cell walls, facilitating the release of bioactive constituents and enhancing antibacterial activity. In contrast, MAE appeared less effective, likely due to rapid heating, which may cause partial degradation of heat‐sensitive phenolic compounds.

Overall, *Vitex agnus‐castus* extracts exhibited stronger antibacterial activity against Gram‐positive bacteria, with particularly notable effects against *S. pneumoniae* and MRSA. However, their efficacy remained lower than that of the reference antibiotic. Nevertheless, these findings support further investigation of the bioactive potential of *Vitex agnus‐castus* and underscore the need for additional studies focusing on the isolation of active compounds, evaluation of potential antibiotic potentiation or enhancement, and elucidation of the underlying mechanisms of action.

## 4 Conclusion

The present study demonstrated that both MAE and UAE, when optimized by RSM, are effective techniques for extracting antioxidant and antimicrobial compounds from *Vitex agnus‐castus*. The selection of ChCl:TetEG:H_2_O for MAE and ChCl:TriEG:H_2_O for UAE proved suitable based on extraction efficiency and solvent properties. Comparative analysis revealed that antioxidant activities and phenolic profiles varied depending on plant part, extraction method, and solvent type. MAE generally yielded higher extraction efficiencies than UAE, particularly for leaf samples, while seeds showed limited phenolic and flavonoid content, likely due to their high lipid composition. HPLC analysis confirmed the successful extraction of key phenolic acids and flavonoids, with notable differences among extraction conditions. Furthermore, the extracts exhibited measurable antibacterial activity under the applied in vitro conditions. These findings should be interpreted as preliminary evidence of biological activity of the crude extracts and do not establish therapeutic efficacy. Overall, the findings highlight the importance of solvent–method optimization and indicate that DES‐based MAE and UAE may represent sustainable and efficient alternatives to conventional solvents for the extraction of bioactive compounds from *Vitex agnus‐castus*. Beyond process optimization, the present study provides the first comprehensive evaluation of DES‐based MAE and UAE for the extraction of bioactive compounds from *Vitex agnus‐castus*. Compared with previously reported conventional solvent extraction methods, this work demonstrates the applicability of environmentally friendly DES systems in combination with modern extraction techniques while providing a comprehensive assessment of antioxidant activity, phenolic composition, and antibacterial properties. These findings broaden the current understanding of green extraction strategies for *Vitex agnus‐castus* and provide a useful foundation for further chemical characterization, mechanistic investigations, and subsequent biological studies.

## Author Contributions

**Buse Ormancı**: investigation, validation, and formal analysis. **Özge Demir**: writing – review and editing, writing – original draft, software, data curation, and visualization. **Berrak Dumlupınar**: data curation, supervision, and methodology. **Şah İsmail Kırbaşlar**: resources, supervision, and investigation. **Aslı Gök**: resources, supervision, project administration, and conceptualization.

## Conflicts of Interest

The authors declare no conflicts of interest.

## Supporting information




**Supporting File**: cbdv71751‐sup‐0001‐SuppMat.docx

## Data Availability

The data that support the findings of this study are available from the corresponding author upon reasonable request.
