# Supplementary material for: Deep Eutectic Solvent Based Microwave and Ultrasound Assisted Extraction of Bioactive Compounds From Vitex agnus‐castus: Optimization and Antioxidant/Antibacterial Evaluation
Source: Chem Biodivers. 2026 Sep 25;23(9):e71751. doi: 10.1002/cbdv.71751 (PMC13614282; doi:10.1002/cbdv.71751)
Supplement: Supplementary file 1 [file CBDV-23-e71751-s001.docx]

**Fig. 1.** Chromatographic peaks, retention times, and detection wavelengths of the identified phenolic compounds: (a) caffeic acid, (b) syringic acid, (c) p-coumaric acid, (d) ferulic acid, (e) rutin, (f) myricetin, (g) quercetin, and (h) kaempferol.
